# Cognitive Performance Profile in Pediatric Moyamoya Disease Patients and Its Relationship With Regional Cerebral Blood Perfusion

**DOI:** 10.3389/fneur.2019.01308

**Published:** 2019-12-12

**Authors:** Jiaxi Li, Xingju Liu, Dong Zhang, Yan Zhang, Rong Wang, Jing Yuan, Jizong Zhao

**Affiliations:** ^1^Department of Neurosurgery, Beijing Tiantan Hospital, Capital Medical University, Beijing, China; ^2^China National Clinical Research Center for Neurological Diseases (NCRC-ND), Beijing, China; ^3^Center of Stroke, Beijing Institute for Brain Disorders, Beijing, China; ^4^Department of Radiology, Beijing Tiantan Hospital, Capital Medical University, Beijing, China

**Keywords:** moyamoya disease, pediatric, cognitive impairment, arterial spin labeling MRI, correlations

## Abstract

**Object:** Moyamoya disease affects the cognitive function of pediatric patients, and compromised cerebral blood flow might be the potential cause. We aimed to explore the specific correlation between cognitive impairment and regional perfusion status in pediatric moyamoya disease patients.

**Methods:** We prospectively enrolled consecutive pediatric moyamoya disease patients admitted to Beijing Tiantan Hospital from July 2017 to March 2019. Arterial spin-labeling magnetic resonance and the Wechsler Intelligence Scale for Children (the 4th edition) were performed on all participants. The cognitive performance of patients was analyzed, and its correlation to cerebral perfusion status was also investigated in the region of interest-based analysis.

**Results:** A total of 21 patients met the inclusion criteria (mean aged 11.14 ± 2.82, male: female = 11:10). Six patients (28.6%) showed no cognitive deficits in any index score, while 15 (71.4%) showed cognitive deficits with differing severity. Nine (42.9%) patients showed overall cognitive impairment, and all cognitive index scores except for Verbal Comprehension Index were significantly lower than the mean scores of normative data with corresponding age. Perceptual Reasoning Index (*p* = 0.019) were statistically lower in patients with radiologically confirmed cerebral infarction. Suzuki stage of the left hemisphere negatively correlated to Full-scale Intelligence Quotient (*r* = −0.452, *p* = 0.039). Region of Interest analysis showed that cerebral blood flow of the left temporal lobe independently associated with the Processing Speed Index (β = 0.535, *p* = 0.041).

**Conclusion:** Pediatric moyamoya disease patients exhibited different levels of cognitive impairment. Cerebral infarction is related to poorer perceptual reasoning ability. Cerebral blood flow in the left temporal lobe positively correlates with processing speed.

## Introduction

Moyamoya disease (MMD) is a cerebrovascular disease characterized by idiopathic stenosis or occlusion of the end of internal carotid arteries (1, 2). Impaired cognitive function is recognized in both adult and pediatric MMD patients (3), and has been hypothetically ascribed to chronic hypoperfusion in adult MMD patients (4). However, the presence of this phenomenon in pediatric patients has been poorly reported.

Single-photon emission computed topography (SPECT) and perfusion CT are widely adopted to evaluate cerebral hemodynamics of MMD patients. However, these methods are radioactive and potentially hazardous, especially for pediatric patients (5, 6). Arterial spin-labeling magnetic resonance imaging (ASL-MRI) is a novel technique that uses labeled intraarterial blood as the contrast substance to acquire perfusion images, with the radiation-free nature being ideal for children (7, 8). Several studies had adopted this technique to evaluate surgical effectiveness in both adult and pediatric MMD patients (9, 10). The application of ASL would facilitate the research about cognition in pediatric MMD.

We hypothesize that cognitive deficit is related to compromised cerebral perfusion in pediatric MMD patients. Hence, we combined ASL technique with the Wechsler Intelligence Scale for Children, the 4th edition (WISC-IV), which could comprehensively evaluate multiple aspects of cognitive impairment (11). This study aimed to delineate the characterized cognitive impairment profile of pediatric MMD patients, and to better understand the correlation between cerebral perfusion in specific regions, and particular aspects of cognitive impairment.

## Materials and Methods

### Participants

Consecutive pediatric MMD patients who were admitted to Beijing Tiantan Hospital from July 2017 to March 2019 and met the inclusion criteria were prospectively enrolled in the current study. The inclusion criteria were as follows: (1) Patients were aged between 6 and 16 years old; (2) Patients did not accept previous revascularization surgery; (3) Patients were cooperative to WISC-IV examination. The diagnosis of MMD was confirmed by digital subtraction angiography (DSA) or magnetic resonance angiography (MRA) based on a 2012 guideline on MMD (12), and only bilateral MMD were included. Patients with incompliance in WISC-IV examination, which may have influenced the reliability of the result, were excluded from the study. The study was performed according to the guidelines of the Helsinki Declaration and was approved by the ethics committee of Beijing Tiantan Hospital. Written informed consent was obtained from all parents of patients, and oral assents were obtained from all patients.

### Clinical Characteristics Evaluation

Clinical characteristics were collected prospectively on admission. Onset manifestations included unilateral or bilateral numbness, weakness, headache, loss of consciousness, and seizure. Based on a pathophysiological basis of these symptoms, patients were classified into hemorrhage, transient ischemic attack (TIA), and cerebral infarction types. Patients with symptoms not attributed to a pathophysiology aforementioned were classified as other types. Age at onset, and the time since the first or the last clinical manifestation until radiological evaluation in this study were analyzed, respectively. Cerebral infarction was recognized on the T2WI FLAIR MR images, and the evaluations were performed by two independent neurosurgeons. The angiographic evaluation was based on DSA or MRA to reflect the severity of angiopathy. Suzuki Stage of each hemisphere was assessed by two independent neurosurgeons blinded to the cognitive performance of the participants. Final assessments were discussed if discrepancies appeared.

### MRI Acquisition

A 3.0T MRI scanner (GE Discovery 750, GE Healthcare, Milwaukee, WI, USA) was used to perform all MR examinations. A 3D stack spiral fast spin echo sequence was used to obtain ASL perfusion maps, and the following parameters were adopted: labeling duration 1,450 ms, post-labeling delay 2,025 ms, field of view (FOV) 200 ×200 mm, slice thickness 4.0 mm, TR 4,762 ms, and TE 10.6 ms.

### ASL Postprocessing

CBF images were normalized to Montreal Neurological Institute (MNI) space using SPM8 software (version 4252; the Wellcome Center for Human Neuroimaging, UK) following a previously reported method (13). To eliminate physiological variations and scan inconsistency, the CBF images were further standardized with a cerebellum standardization method to obtain a relative CBF (rCBF) value (14, 15). Briefly, the CBF value of each voxel was standardized by dividing the mean CBF extracted from cerebellum of the same patient since the perfusion of cerebellum is seldom affected in MMD patients. According to the Talairach Daemon database brain atlas (16), we extracted mean rCBF of ROIs of frontal, parietal, temporal, and occipital lobes of each hemisphere for ROI-based analysis. Image illustration of ROIs were presented in Supplementary Figure 1.

### Wechsler Intelligence Scale for Children

All patients enrolled received an intelligence evaluation based on the Mandarin version of WISC-IV. The results of this test consist of the Full-scale Intelligence Quotient (FSIQ) that indicates overall intelligence, and four index scores indicating different aspects of cognitive ability, including Verbal Comprehension Index (VCI), Perceptual Reasoning Index (PRI), Working Memory Index (WMI), and Processing Speed Index (PSI).

All these index scores were calibrated with normative samples of the same age. Cognitive impairment was defined as an index lower than 85 which represents a standard deviation less than the mean score of the normative data. Levels of cognitive impairment were defined as follow: 70 < index < 85: mild; 55 < index < 70: moderate; index < 55: severe impairment.

### Statistical Analysis

Two-tailed Student's *t*-test was adopted to compare WISC-IV index scores of patients to mean scores of the normative data. The relationship between clinical characteristics and indexes derived from WISC-IV were tested using Pearson's correlation test (for continuous variables), two-tailed Student's *t*-test (for dichotomized variables) or one-way analysis of variance (for categorized variables) as appropriate.

In ROI-based analysis, the correlation between relative CBF in each ROI and cognitive index scores were tested using Pearson's correlation test. In order to eliminate the influence of confounding factors, multivariate regression models were constructed for variables showing significant correlations in univariate analysis. Variance inflation factors of variables were calculated to evaluate collinearity. Standardized coefficients (β) of variables are presented.

Continuous variables are presented as the means ± standard deviations, and categorical variables are presented as counts (with percentage). Statistical analysis was performed using the R statistical program (version 3.6.0; R core team), and a significance level of *p* < 0.05 was recognized as significant.

## Results

### Clinical Characteristics

During Sep 2017 to Mar 2019, 21 pediatric MMD patients who met the criteria were enrolled in the current study. All patients underwent ASL-MRI examination, WISC-IV evaluation, and angiography (either DSA or MRA). The mean age of the patients enrolled was 11.14 ± 2.82 years old, and the male to female ratio was 11: 10. Of these participants, 5 (23.8%) manifested hemorrhage at onset, 3 (14.3%) manifested cerebral infarction, 11 (52.4%) manifested TIA, and 2 (9.5%) experienced other onset symptoms. The mean age at onset was 9.62 ± 3.01 years, with a mean symptom duration of 19.33 ± 24.07 months. Suzuki stage ranged from 1 to 5, and posterior circulation was not involved in all participants. Detailed basic characteristics of patients are listed in Table 1.

**Table 1:** Basic characteristics.

**Variables**	***N* = 21**
Age	11.14 ± 2.82
Female sex	10 (47.6)
Age at disease onset	9.62 ± 3.01
Duration of symptoms (Months)	19.33 ± 24.07
Time since last onset (Months)	7.42 ± 17.70
**Episodes of onset**	
One episode	10 (47.6)
2–4 episodes	7 (33.3)
≥5 episodes	4 (19.0)
**Onset types**	
Hemorrhage	5 (23.8)
Cerebral infarction	3 (14.3)
Transient ischemic attack	11 (52.4)
Other	2 (9.5)
Previous cerebral infarction	6 (28.6)
**Neurological status (mRS)**	
0	18 (85.7)
1	2 (9.5)
2	1 (4.8)
**Suzuki stage of left hemisphere**	
I	0 (0)
II	5 (23.8)
III	9 (42.9)
IV	6 (28.6)
V	1 (4.8)
VI	0 (0)
**Suzuki stage of right hemisphere**	
I	4 (19.0)
II	2 (9.5)
III	5 (23.8)
IV	8 (38.1)
V	2 (9.5)
VI	0 (0)

*Values are numbers of cases (%) unless otherwise indicated. Mean values are presented with SDs*.

### WISC-IV Evaluation

**Figure 1:**
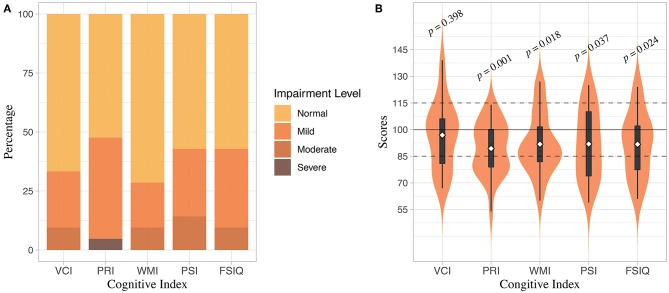
Distribution of WISC-IV Index scores. **(A)** Distribution of cognitive impairment severity represented by different index scores. **(B)** Distribution of WISC-IV index scores. Upper and lower edges of black boxes denote the third and first quantile of index scores, and white dots denote means of index scores. *P*-value of *t*-test comparing index scores of participants to the mean scores of normative data were presented.

Among 21 enrolled patients, 6 (28.6%) showed no cognitive deficit in any index score while 15 (71.4%) showed cognitive deficits of different severity. Specifically, 4 (19.0%) showed impairment in a single index score and 11 (52.4%) showed multi-index score impairment. Overall cognitive impairment, represented by an FSIQ <85, was detected in 9 (42.9%) patients. Further detailed distribution of index scores was presented in Table 2 and Figure 1A. Comparing to mean scores of the normative data, all index scores of patients except for VCI were significantly lower (Figure 1B).

**Table 2:** Distribution of index scores.

**Index scores**	**Average**	Normal^*^	Mild impairment^*^	Moderate impairment^*^	Severe impairment^*^
VCI	96.76 ± 20.1	14 (66.67)	5 (23.81)	2 (9.52)	0 (0)
PRI	89.14 ± 15.61	11 (52.38)	9 (42.86)	0 (0)	1 (4.76)
WMI	92.76 ± 18.04	15 (71.43)	4 (19.05)	2 (9.52)	0 (0)
PSI	91.14 ± 20.32	12 (57.14)	6 (28.57)	3 (14.29)	0 (0)
FSIQ	91.71 ± 19.44	12 (57.14)	7 (33.33)	2 (9.52)	0 (0)

* *Presented as number of cases (with percentage of all participants). Mild impairment: score <85; moderate impairment: score <70; severe impairment: score <55*.

### Relationships Between Clinical Characteristics and WISC-IV Indexes

**Figure 2:**
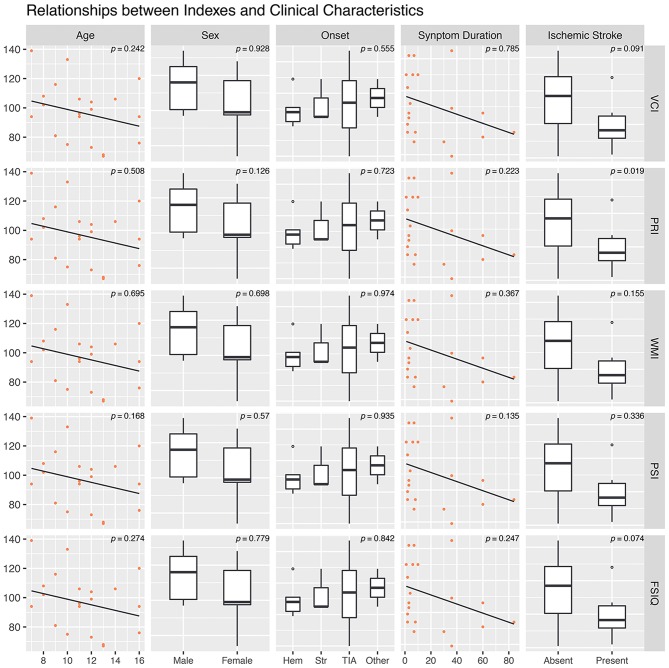
Relationships between Clinical Characteristics and Cognitive Index scores.

**Figure 3:**
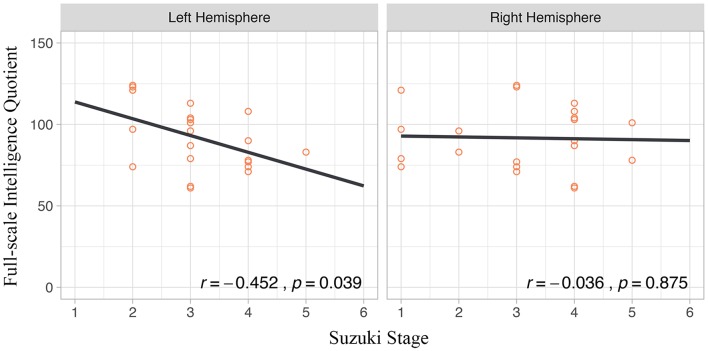
Correlations between Full-scale Intelligence Quotient and Suzuki Stage.

Correlations between age, symptom duration, and 5 WISC-IV index scores were tested, and no statistically significant correlation was found. Distributions of these indexes in different sex, onset manifestation, and presence of cerebral infarction were tested as well. Only PRI (*p* = 0.019) were statistically lower in MMD patients with radiologically confirmed cerebral infarction (Figure 2). Suzuki stage of the left hemisphere showed a significant correlation with FSIQ (*r* = −0.452, *p* = 0.039), whereas Suzuki stage of the right hemisphere did not exhibit significant correlation (*p* = 0.875) (Figure 3).

### Spatial Distribution of CBF

**Figure 4:**
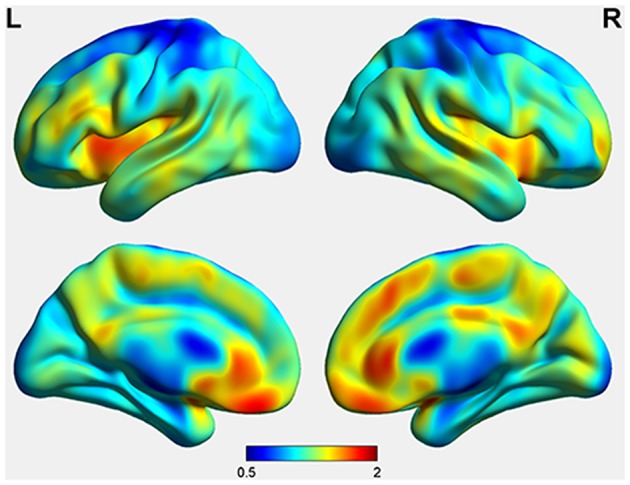
Spatial Distribution of Cerebral Blood Flow of Pediatric Moyamoya Disease Patients. Values were standardized by dividing cerebellar CBF for each individual and then collectively analyzed.

CBF relative to cerebellum was averaged across all participants and the spatial distribution of CBF was projected to Ch2 brain model (Figure 4). Precentral gyri and postcentral gyri paracentral lobules of both hemispheres showed relatively lower CBF, while anterior, medial, and posterior cingulate cortex, as well as precuneus, showed relatively higher CBF.

### ROI Based Analysis

**Figure 5:**
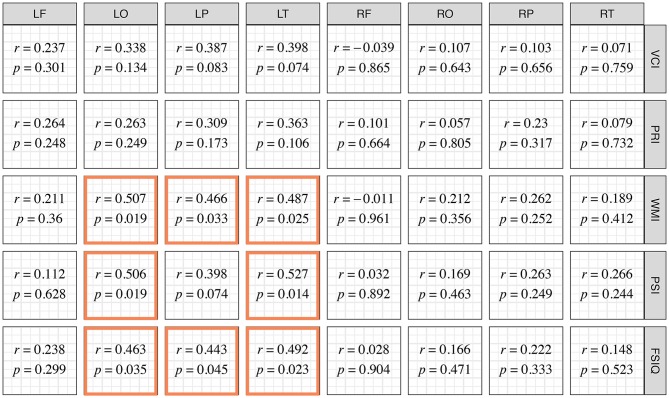
Correlation Coefficient between Lobular Relative Cerebral Blood Flow and Index Scores of WISC-IV. Correlations with statistical significance are emphasized with colored frames.

Correlation between mean CBF from each ROI and different WISC-IV index scores were tested. Among these relationships, correlations between regional CBF of left temporal, parietal, and occipital lobes, and WISC-IV indexes including WMI, PSI, and FSIQ were statistically significant (Figure 5).

These significant correlations were further evaluated in multivariate linear regression models adjusted for sex, onset type, symptom duration, and the ipsilateral Suzuki stage. Variance inflation factors of variables in all the models were <4. Among these models, the CBF in left temporal lobes significantly associated with PSI (β = 0.535, *p* = 0.041) (Table 3).

**Table 3:** Association between Regional CBF and WISC-IV Scores.

	**WMI**		**PSI**		**FSIQ**	
	β^*^	***p*-value**	β^*^	***p*-value**	β^*^	***p*-value**
Left temporal	0.484	0.068	0.535	0.041	0.460	0.062
Left parietal	0.366	0.179	0.329	0.232	0.307	0.232
Left occipital	0.563	0.096	0.490	0.154	0.491	0.124

* *Standardized regression coefficients (β) were presented*.

*All coefficients were adjusted by sex, onset type, symptom duration, and ipsilateral Suzuki stage*.

## Discussion

Growing attention has been paid to cognitive impairment in MMD. Although research in adult patients has made substantial achievements, cognitive impairment in pediatric MMD patients has not been deeply investigated. To our knowledge, this is the first study combining ASL-MRI technique and cognitive function investigation in pediatric MMD patients. Our results demonstrated that 71.2% (15 in 21 patients) of participants exhibited different levels of cognitive impairment, and average cognitive performance is significantly poorer than normative samples of corresponding age in most index scores. History of cerebral infarction is linked to lower PRI. ROI-based study identified that the CBF in the left temporal lobe was independently associated with PSI.

Both adult and pediatric MMD patients had been reported to suffer from cognitive impairment (3), but cognitive decline that occurred in childhood was believed to cause intellectual disability and lead to social adaptation difficulty when they reach adulthood, which is more influential to the quality of life (2, 17). Although cognitive functioning in pediatric patients is considered important, related research was scarce and poor in quality due to either obsolete psychological evaluation tools or small sample size (18–21). We adopted WISC-IV to evaluate the cognitive function of participants (11). WISC-IV, an upgraded version of WISC-III, has been widely adopted in most recent research and provides a more comprehensive cognitive assessment with more specified cognitive abilities compared to WISC-III (22). Etiology and pathophysiology of MMD are complicated, so a detailed cognitive profile could facilitate the identification of specific mechanisms leading to cognitive impairment. Our study showed that a considerable portion of participants exhibited cognitive decline compared to normative samples, and the compromised cognitive profile varied among patients. In our study, 28.5% patients had working memory impairment and 42.9% had processing speed deficit, which is higher than a previous report (21). The relatively small sample size and different standards to define cognitive deficits may contribute to this discrepancy. 42.9% participants were found with under-threshold FSIQ in our study, in line with a previous report that showed 37% of pediatric MMD presented compromised overall intelligence (23). Although not all pediatric patients suffer from cognitive problems, it is still valuable finding out related factors associated with cognitive impairment. This would further guide the treatment strategy of MMD.

Researchers have discussed potential indicators of cognitive impairments of pediatric MMD patients. Longer duration of symptoms, cerebral infarction, and bilateral involvement had been suggested to associate with poorer cognitive performance (21, 24–26). However, these associations were not significant in a meta-analysis study (3). In our study, only radiologically-evident cerebral infarction was associated with lower cognitive index scores. Theoretically, with the prolonged duration of chronic hypoperfusion, the cognitive impairment would be exacerbated. Besides, MMD patients with hemorrhagic onset were suggested to suffer less from hypoperfusion compared to those with ischemic onset (6); therefore, cognitive deficiency level might be discrepant between two types of patients. Nevertheless, our results did not support this hypothesis. Relatively small sample size may be one possible cause, and other confounding factors such as hemodynamic status may contribute too.

Correlation between cerebral blood perfusion and cognitive function in adult MMD patients has been investigated, and hypoperfusion in several specific brain regions had been identified as responsible for corresponding cognitive deficits. Both cerebral vascular reserve and CBF in the frontal lobe were proved to correlate to executive functioning in adult MMD patients (4, 15). Moreover, the associations between CBF in several brain areas, including left ventral middle cerebral artery territory and bilateral dorsal anterior cerebral artery territory, and multiple aspects of cognitive function had also been reported (27). In pediatric patients, only one study directly discussed the correlation between CBF and cognitive dysfunction, which proposed a tendency that patients with lower intelligence scores showed poorer cerebral perfusion (18). ASL is a novel technique for cerebral hemodynamics evaluation without radiation and extrinsic contrast reagents, which has been adopted in clinical use recently (8). It exhibited a satisfactory efficacy in MMD patients compared to SPECT (24). Exerting this advantage, we adopted ASL for CBF assessment in our pediatric participants. In our study, we identified positive correlations between cognitive performance and regional cerebral blood flow. The severity of MMD, represented by the Suzuki stage, is a crucial part of clinical evaluation. Our results also showed that the Suzuki stage of the left hemisphere significantly correlated with overall cognitive performance. A similar relationship was reported in adult MMD patients previously (28). Interestingly, only the left hemisphere exhibited this relationship, concordant to relationships between cerebral perfusion and cognitive performance. Progressive compromise of cerebral hemodynamics accompanying the advancement of the Suzuki stage may be responsible for this relationship (29), since all of the participants in this study were right-handed, which means the left hemispheres were dominant hemispheres. Eloquent cortex was believed to be located in the temporal and the frontal lobe of the dominant hemisphere; therefore, impaired cognitive performance could be attributed to the impact of hypoperfusion on language processing. Moreover, we hypothesize that the advanced cognitive process itself could have a lateralized anatomical basis similar to language processing. Revascularization surgery was suggested to prevent further cognitive impairment in pediatric MMD patients, or even to improve this deficit (18, 20, 25). Increased mean CBF was reported to associate with intelligence improvement (18). Based on our results, however, the CBF increase in the revascularized regions is hypothesized to be a more direct mechanism. Therefore, the preoperative evaluation of cerebral hemodynamics would facilitate selected revascularization, and it would further reinforce the therapeutic effect on cognitive deficits.

Anatomical location of advanced cognitive function has undergone intensive investigation. Despite a few studies reporting a correlation between hemodynamic compromise in temporal lobe and cognitive impairment (26, 30), more researchers found that regions in the frontal lobe are linked to executive function, both from aspects of hemodynamics and metabolism (4, 15, 31, 32). Using multivariate linear regression to adjust for confounders, we found that the average CBF of the left temporal lobe independently associated with processing speed. No significant correlation was found between CBF in the frontal lobe and cognitive performance. The small sample size could lead to this discrepancy. On the other hand, the majority of research reporting the relation between the frontal lobe and cognitive function in MMD focused on adults, even the elderly. Whether the anatomical basis of cognition in children is different still needs deeper investigation.

Some potential limitations exist in our study. First, the number of patients enrolled still limits the validity of the results. More convincing conclusions would be drawn after the cohort develops. Second, since it is suggested that repeated WISC-IV should be performed after a 2-year interval to avoid the influence caused by the learning effect in the first examination, we did not incorporate a follow-up investigation in the current study. Improvement of cerebral perfusion and cognitive performance after revascularization will be evaluated in future studies.

## Conclusion

This study demonstrates that 71.2% (15 in 21 patients) of pediatric MMD patients exhibited different levels of cognitive impairment, and average cognitive performance is significantly poorer than the normative samples of corresponding age. The history of cerebral infarction is linked to poorer perception and general cognitive ability. Furthermore, we identified that the average CBF in the left temporal lobe independently associated with processing speed and cognitive proficiency.

## Data Availability Statement

The datasets generated for this study are available on request to the corresponding author.

## Ethics Statement

The studies involving human participants were reviewed and approved by The ethics committee of Beijing Tiantan Hospital. Written informed consent to participate in this study was provided by the participants' legal guardian/next of kin.

## Author Contributions

JL and XL: conception, design, analysis, and interpretation of data. JL, XL, and JY: acquisition of data. JL: drafting the article. XL, YZ, DZ, RW, JY, and JZ: technical, administrative supports, and surgery. JZ: approving the final version of the manuscript on behalf of all authors and study supervision. All authors critically revising the article and reviewing submitted version of manuscript.

## Conflict of Interest

The authors declare that the research was conducted in the absence of any commercial or financial relationships that could be construed as a potential conflict of interest.

## Supplementary Material

Supplementary Figure 1 Illustration of lobular ROIs used for regional cerebral blood flow extraction. ROIs of the two hemispheres were symmetrical. Only the ROIs of the left hemisphere were presented. **(A)** Frontal lobe; **(B)** parietal lobe; **(C)** occipital lobe; and **(D)** temporal lobe.

## Abbreviations

MMD: moyamoya disease
SPECT: single photon emission computed topography
ASL: arterial spin-labeling
WISC-IV, Wechsler Intelligence Scale for Children: the 4th edition
DSA: digital subtraction angiography
MRA: magnetic resonance angiography
FSIQ: full-scale intelligence quotient
VCI: verbal comprehension index
PRI: perceptual reasoning index
WMI: working memory index
PSI: processing speed index.
