# Supplementary figures and images for: Cognitive Performance Profile in Pediatric Moyamoya Disease Patients and Its Relationship With Regional Cerebral Blood Perfusion
Source: Front Neurol. 2019 Dec 12;10:1308. doi: 10.3389/fneur.2019.01308 (PMC6920207; doi:10.3389/fneur.2019.01308)

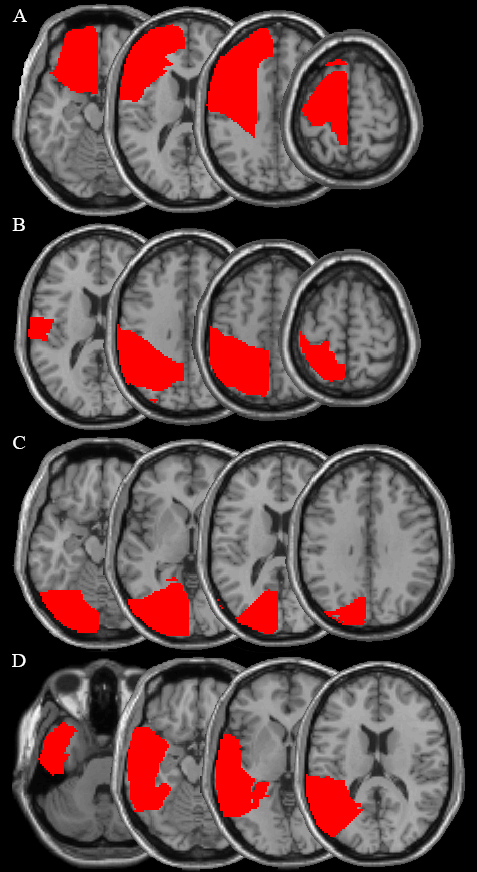

Supplement: Supplementary Figure 1 — Illustration of lobular ROIs used for regional cerebral blood flow extraction. ROIs of the two hemispheres were symmetrical. Only the ROIs of the left hemisphere were presented. (A) Frontal lobe; (B) parietal lobe; (C) occipital lobe; and (D) temporal lobe. [file Image_1.TIF]
